# Arthrosia Atonica

**Published:** 1829-07-01

**Authors:** 


### XIII.

### BALTIMORE INFIRMARY.

### ARTHROSIA ATONICA.

[Dr. T. H. Wright, Reporter.]

We recommend to the serious attention of our surgical brethren, the following report, drawn up with a modesty, candour, and ability which do great honour to the reporter. We hope some of our metropolitan REPORTERS will imitate the example set them by Dr. Wright.

*Case of Arthrosia Atonica.*—"Elizabeth Henry, aged thirty-five, admitted into the Baltimore Alms-house 1st of May, 1828. She is of middle size, and well-formed, with black hair and eyes, and fair complexion; says she had led an active, temperate life, enjoyed good health, and borne children. When admitted, she could not stand without support, and was unable to walk, from pain in the upper half of the right thigh, and weakness of that limb. The day after admission she had no fever or headache, pulse soft, slow, and equal; skin cool, no cough, tongue clean, appetite good, bowels regular, sleep natural, except from occasional spasms of the thigh. Thus the circulating, cerebral, respiratory, digestive, and nervous systems, wholly out of fault. The complaint of the patient was referred by herself entirely to the thigh, and a point in the front middle portion of the limb indicated as the seat of predominant sensibility. The patient stated that she was attacked suddenly, fourteen days before her admission into the Alms-house, by acute pain of the thigh, and from the moment of attack had been unable to walk.

"The thigh now exhibited no mark of disease. There was no heat, inflammation, tension, or enlargement; the skin and muscles soft and pliable, and the part not tender to the touch, except pressed toward the centre of the limb, when there was shrinking and complaint. The hip was neither swelled, nor super-sensitive, even to firm pressure, and the whole limb, compared with the opposite one, betrayed no visible mark of difference. On my pronouncing the apparent absence of local disease, the patient said the pain was in the bone. I confess frankly that I regarded this poor woman as either ignorantly or wilfully exaggerating a common affair of chronic rheumatism.

"The patient had taken some medicine, and the part had been freely blistered, (without relief,) before her admission into the Alms-house. A gum pill, which has produced in the institution much relief from chronic rheumatism, composed of guaiacum, camphor, and hyosciamus, was directed for the patient, with anodyne embrocation of the part. I saw this case every other day for a fortnight, during which time its circumstances did not sensibly alter. The patient continued without general indisposition, preserving a quiet pulse, cool skin, clean tongue, and good appetite, the limb retaining the appearance and state before described: she expressed herself relieved from pain by the pills, and asked to have them renewed when out. On the 16th of

May she requested me to look again at her limb, stating that it had been severely painful the night before, and that it was now shorter than the other. The thigh was examined, and exhibited its usual appearance; on comparing the lower extremities, the position of the right leg was natural, but the bottom of the foot presented on a line with the internal malleolus of the other leg; that is, the right limb appeared about two inches shorter than the left. After every attention to the equipoise of the pelvis, and the full, but easy, extension of the limb, the right foot rested an inch and a half higher than its fellow. The hip was next carefully examined. The dorsum ilii was free, and the head of the femur could not be felt. These circumstances, and the natural position of the limb, established that there was no dislocation. But there might be something as bad, and on gently extending, raising, and rotating the limb, a bold, rough crepitation was as distinctly felt as in free recent fracture. The head of the femur, or the acetabulum, or both, were gone, and the limb had been drawn up by the muscles in its natural axis.

"She is at present, (four weeks after destruction of the joint,) able to sit up, and even walks a little by support of one crutch. Her appearance betrays no mark of impaired health, and there is no longer pain or soreness in any part of the limb. In her case, too, the only inconvenience is shortening, and disability of movement; the injured limb is two inches short, and can be advanced or retracted by its own muscles only a very limited space. The aspect, or presentation of the limb is natural; in standing, sitting, or lying, there is neither inversion nor eversion of the foot. It may be noticed here that at one period, soon after the articulating surfaces were destroyed, the limb was found to have lost its proper attitude, and to lay upon the other in a state of complete inversion. It was easily restored to its natural aspect, and by restricting the patient to a position on her back for some time, it did not again decline from its proper axis.

"This is a melancholy case, and suggests a lesson of modesty of useful import; namely, to distrust our sagacity in forming a judgment of obscure affections. To myself at least, two such cases occurring nearly together, in neither of which the terrible tendency of the disease was fully apprehended, until revealed by its consummation, plainly admonish the necessity of close, careful, and circumstantial investigation in such cases, as well as the propriety of proceeding on the principle of guarding against the worst that might happen, even though the danger of that result is not plainly apparent. I cannot charge myself with neglect or slight of duty in this case; the countenance, general health of the patient, and the external state of the parts deceived me, and were calculated to deceive.* But the thigh and hip were frequently and carefully examined. Not a trace of inflammation, swelling, or soreness to the touch existed in the soft parts, either at first or subsequently. The poor woman herself was right: she constantly declared that the disease was in the bone, and this is not the first time a patient's sensations have been more faithful than the tact of the profession.

"All I learned further than has been already told, was that the pain commenced with a 'sudden dart,' in the front of the thigh, two inches below the trochanter, while the woman was standing in market. On the first days of pain, a few vesications formed on the surface of the thigh, about the seat of the pain, but these soon subsided and left the part free from any after appearance of disease. The patient complained for the first time on the 16th of May, (the day after the joint broke up,) of pain at the top of the knee, which continued severe for many days.

"The total absence of constitutional concern in a work of local destruction

* "It is a state of things in fact, not easy of detection, and even if discovered, perhaps still less susceptible of safe conduct. There is little reason to conclude that after admission into the infirmary, the final consequence could have been averted by any mode of management."

of so serious a character, is an unusual phenomenon, fitted to mask the nature of the disease, and divert from calculation on an issue of which there existed so few and equivocal admonitions. When this woman was admitted into the institution, pain of the right thigh not constant, nor very acute, with loss of motive power in the limb, constituted the only marks of disease, nay, the only evidence of decline from the most perfect state of health. After entering the infirmary the case did not at any time present even an ephemeral feverish tumult; the skin was always cool, and pulse temperate. The pain, or rather uneasiness of the part, though seldom wholly absent, was never distressing in the day, and was readily averted or mitigated by an anodyne at night. The ultimate destruction of the joint, though attended by extreme distress of feeling, produced scarcely a sensible febrile movement, and the faint disturbance of circulation which attended the process in question ceased very soon, and left the patient with a pulse calm and quiet, as in health.

"The condition of the limb itself is not less remarkable than the constitutional circumstances just adverted to. It has not been, and is not even now, (7th of June,) at all wasted or altered in form. Its state as to size, temperature, and all circumstances, shortening alone excepted, is perfectly natural, and there is no longer pain of the part during a state of strict repose. This woman now gets out of bed, (to make necessary evacuations,) and accomplishes the task by lifting the thigh and leg off the bed with both her hands. She says that when she is up resting on the sound limb, the affected leg and thigh turn uniformly of a very dark, or in her language, black colour."*

We solicit the attention of hospital surgeons to this case, and should be glad to learn the sentiments of the experienced members of the surgical profession on it.—*Ed.*

* Amer. Journ. of the Med. Sciences, No. V.

